# Site-2 Protease Slr1821 Regulates Carbon/Nitrogen Homeostasis during Ammonium Stress Acclimation in Cyanobacterium *Synechocystis* sp. PCC 6803

**DOI:** 10.3390/ijms24076606

**Published:** 2023-04-01

**Authors:** Shiqi Lin, Shiliang Li, Tong Ouyang, Gu Chen

**Affiliations:** School of Food Sciences and Engineering, South China University of Technology, 381 Wushan Road, Guangzhou 510641, China

**Keywords:** transcriptomics, nitrogen uptake and assimilation, ammonium integration, PII, PirC, RcbR

## Abstract

Excess ammonium imposes toxicity and stress response in cyanobacteria. How cyanobacteria acclimate to NH_4_^+^ stress is so far poorly understood. Here, *Synechocystis* sp. PCC6803 S2P homolog Slr1821 was identified as the essential regulator through physiological characterization and transcriptomic analysis of its knockout mutant. The proper expression of 60% and 67% of the NH_4_^+^ activated and repressed genes, respectively, were actually Slr1821-dependent since they were abolished or reversed in ∆*slr1821*. *Synechocystis* 6803 suppressed nitrogen uptake and assimilation, ammonium integration and mobilization of other nitrogen sources upon NH_4_^+^ stress. Opposite regulation on genes for assimilation of nitrogen and carbon, such as repression of nitrogen regulatory protein PII, PII interactive protein PirC and activation of carbon acquisition regulator RcbR, demonstrated that *Synechocystis* 6803 coordinated regulation to maintain carbon/nitrogen homeostasis under increasing nitrogen, while functional Slr1821 was indispensable for most of this coordinated regulation. Additionally, *slr1821* knockout disrupted the proper response of regulators and transporters in the ammonium-specific stimulon, and resulted in defective photosynthesis as well as compromised translational and transcriptional machinery. These results provide new insight into the coordinated regulation of nutritional fluctuation and the functional characterization of S2Ps. They also provide new targets for bioengineering cyanobacteria in bioremediation and improving ammonium tolerance in crop plants.

## 1. Introduction

Cyanobacteria are a group of photosynthetic prokaryotes that contribute to primary production on a global scale. They contribute a significant portion to the oxygen yield and play important roles in biogeochemical cycles, especially in the global carbon and nitrogen cycles. In addition to their ecological importance, due to their photosynthetic lifestyle, cyanobacteria also arouse interest as hosts for the sustainable production of biofuel and high-value chemicals, as well as bioremediation of environmental pollutants [1]. However, both fundamental research and biotechnological application are hampered by limited knowledge about the regulation of metabolic fluxes in these organisms [2,3]. In their natural environment, these organisms are exposed to fluctuating nutrient conditions. As one of the earliest branching groups of organisms on our planet, cyanobacteria are subjected to various selective pressures during their long history of evolution. They settle down in a variety of environments and have evolved various acclimation mechanism to stress and fluctuating levels of nutrients [4,5]. Carbon and nitrogen are the two most abundant nutrient elements for all living organisms; thus, carbon/nitrogen homeostasis control is the key point for the maintenance of cellular homeostasis. Upon nitrogen starvation, cyanobacteria apply a sophisticated signal transduction network sensing the metabolites such as 2-oxoglutarate and cAMP to maintain C/N homeostasis, while essential sensors and regulators have been discovered, including transcription factor NtcA, the versatile PII signalling protein and PII-interactor PirC, etc. [6,7,8,9,10]. However, limited research has been reported about cyanobacteria acclimation to nitrogen surplus, especially high concentration of NH_4_^+^, which could not be merely the reverse process of nitrogen starvation, partly due to the ammonium toxicity [11].

The intensive application of NO_3_^−^- and NH_4_^+^-based fertilizers in croplands, feeding-animal fecal deposition and dye industry effluent have caused global nitrogen pollution, including the eutrophication of water reservoirs, soil contamination and atmospheric pollution, which are recognized as a serious worldwide issue of public and economic concern [12,13,14,15]. Synergy of bioremediation of high nutrients containing wastewater with potentially valuable biomass production through microalgae including cyanobacteria cultivation is an economical and sustainable approach to mitigate nitrogen pollution [16,17,18]. However, the ammonium acclimation mechanism in cyanobacteria was still a mystery. Since cyanobacteria are the ancestors of plastids in plants, the ammonium toxicity and tolerance mechanism in plants were consulted. Ammonium triggers rapid changes in plant cellular pH, gene transcription and post-translational modifications, leading to coordinated ammonium uptake and assimilation [19], altered oxidative and phytohormonal status [20,21], excess proton accumulation in plastids [22] and reshaped roots and shoots [23]. Intriguingly, the *Arabidopsis* plastid AMOS1/EGY1 was discovered to regulate global gene expression response to ammonium stress [24]. AMOS1/EGY1 is the metalloprotease S2P (site-2-protease) homolog required for chloroplast development [25,26]. S2Ps are a conserved family of intramembrane proteases that cleave transmembrane substrates to regulate signal transduction and maintain proteostasis [27,28]. Eukaryotic S2Ps, including human S2Ps, perform intramembrane proteolysis of transcription factor precursors to regulate signal transduction for lipid metabolism and endoplasmic reticulum stress responses, while *E.coli* S2P cleaves anti-sigma factor to free sigma factor in the extracytoplasmic stress response [27,28]. Thus, the effect of S2P regulation is usually prominent at the transcriptional level. Consistent with their prevalence throughout all three domains of life, S2P homologs are widely spread in the cyanobacteria phylum, suggesting the essential role of this group of proteins [29]. Cyanobacterium *Synechocystis* sp. PCC 6803 (hereafter referred to as *Synechocystis* 6803) has four S2P homologs, and several of them were involved in stress response, such as Slr0643 and Sll0528 in acid and salt stress acclimation, respectively [30,31]. However, their involvement in ammonium acclimation has not been explored so far.

This study aimed to clarify the role of the S2P homolog Slr1821 in ammonium acclimation. Our results indicate that *Synechocystis* 6803 coordinate expression to maintain carbon/nitrogen homeostasis upon NH_4_^+^ stress in an Slr1821-dependent manner. It provides new insight into the coordinated regulation to maintain C/N homeostasis during nutritional fluctuation and the functional characterization of photosynthetic S2P homologs, as well as lays the foundation for bioremediation of high-ammonium-containing wastewater using cyanobacteria.

## 2. Results and Discussion

### 2.1. Functional Slr1821 Is Essential to the Effective Acclimation of Synechocystis 6803 to High Concentration of NH4^+^

**Figure 1:**
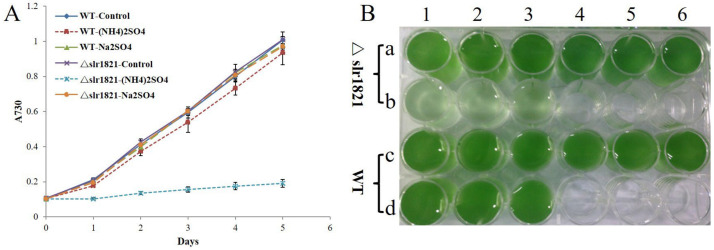
Functional Slr1821 is essential to the successful acclimation of *Synechocystis* 6803 to high concentration of NH_4_^+^. (**A**) Wild type (WT) and ∆*slr1821* knockout mutant were grown in BG11 medium supplemented with 15 mM (NH_4_)_2_SO_4_ at 29 °C under constant illumination of 25 μmol·m^−2^·s^−1^. As normal control, WT and ∆*slr1821* were grown in normal BG11 medium. To eliminate the impact of SO_4_^2−^, WT and ∆*slr1821* were grown in BG11 medium with 15 mM Na_2_SO_4_ as background control. (**B**) The phenotype of WT and ∆*slr1821* on the fourth day in (**A**). a1, a2, a3 and c1, c2, c3 were ∆*slr1821* and WT in BG11 medium, respectively. a4, a5, a6 and c4, c5, c6 were ∆*slr1821* and WT in BG11-Na_2_SO_4_, respectively. b1, b2, b3 and d1, d2, d3 were ∆*slr1821* and WT in BG11-(NH_4_)_2_SO_4_, respectively.

In order to explore the physiological functions of Slr1821, a ∆*slr1821* knockout mutant was constructed via homologous recombination with a kanamycin resistance cassette to replace the Slr1821 coding sequence (Supplemental Figure S1). The ∆*slr1821* strain showed similar phenotype with wild type (WT) under normal phototrophic growth conditions, as well as under high light, mixotrophic growth, and salt or ethanol stress. However, significantly retarded growth of ∆*slr1821* was observed upon high concentration of (NH_4_)_2_SO_4_ (Figure 1). The 15 mM (NH_4_)_2_SO_4_ was toxic for most plants, while WT *Synechocystis* 6803 could successfully acclimate to it and such acclimation was compromised in ∆*slr1821*. Using Na_2_SO_4_ as the background control, it was concluded that NH_4_^+^ rather than SO_4_^2−^ was the causal factor for growth inhibition in ∆*slr1821* (Figure 1). Three independent knockout strains of Slr1821 were analyzed and similar results were attained, which confirmed the essential role of functional Slr1821 during acclimation to high concentration of NH_4_^+^.

### 2.2. Global Transcriptomic Comparison of WT and ∆slr1821 Knockout Mutant upon NH_4_^+^ Stress

To investigate the molecular mechanism of Slr1821 in the NH_4_^+^ stress response, RNA sequencing was used to explore the global transcriptomic profiles of ∆*slr1821* and WT under conditions with or without NH_4_^+^ stress for 30 min. For each of the four groups of samples, three replicates were analyzed. About 15,167,632 to 24,438,662 reads were obtained as clean data for the twelve samples, and 3.02 G bases of clean data were obtained for each sample on average (Supplemental Table S1). Saturation curves for twelve samples indicated that the sequencing coverage was appropriate to reveal the expression level of more than 98.7%~99.4% genes (Supplemental Figure S2A). In total, 3653 transcripts were detected. Differentially expressed genes were figured out through the comparison between groups, including the ∆*slr1821*/WT under condition without or with NH_4_^+^ stress, and the comparison with/without NH_4_^+^ stress in either WT or ∆*slr1821* (Supplemental Figure S2B). The screening criteria were set as FDR < 0.05 and |log_2_FC| ≥ 1. The reliability of RNA sequencing data was confirmed through quantitative RT-PCR of representative gene, with Pearson’s correlation coefficient as high as 0.98 (Supplemental Figure S2C).

**Figure 2:**
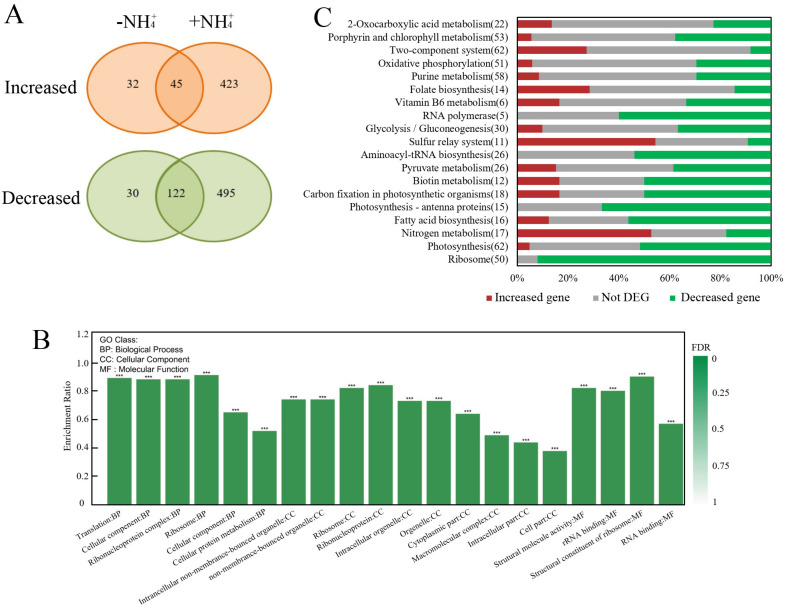
Transcriptional profile in ∆*slr1821* compared with the wild type (WT). (**A**) Total number of genes which were significantly increased or decreased in ∆*slr1821* relative to WT under conditions without or with NH_4_^+^ stress for 30 min. (**B**) The top 20 enriched GO categories in biological process, cellular component and molecular function of differentially expressed genes in ∆*slr1821* compared with WT under NH_4_^+^ stress. The enrichment p value was adjusted through FDR (false discovery rate) and FDR<0.001 are labeled as ***. (**C**) Distribution of the significantly increased or decreased genes in ∆*slr1821* compared with WT under NH_4_^+^ stress in the enriched KEGG categories.

Firstly, effects of the *slr1821* knockout on the transcriptional profiles were analyzed (Figure 2). Compared with WT, 500 genes were expressed at higher levels in ∆*slr1821*, while 647 genes were expressed at lower levels. Among them, 423 (84.6%) higher-expressed genes and 495 (76.5%) lower-expressed genes in ∆*slr1821*/WT were exclusively found under NH_4_^+^ stress. Gene Ontology (GO) enrichment analysis of the differentially expressed genes (DEGs) between ∆*slr1821* and WT upon NH_4_^+^ stress revealed the strongly enriched GO categories such as translation, ribosome and RNA binding for biological process, cellular component and molecular function, respectively. Further detailed distribution of the significantly up- or downregulated genes in enriched KEGG categories indicated that most DEGs of translation (ribosome and aminoacyl-tRNA biosynthesis), photosynthesis and fatty acid biosynthesis were repressed in ∆*slr1821*, while most DEGs in nitrogen metabolism and the sulfur relay system were unusually activated. The unusually activated genes in the sulfur relay system converged on the biosynthesis of molybdenum cofactor (MoCo). MoCo is the essential prosthetic group of nitrate reductase, which together with nitrite reductase accomplish the principal pathway for nitrate assimilation into ammonium. These results implied that under NH_4_^+^ stress, the functional Slr1821 was required for the homeostasis of nitrogen metabolism, photosynthesis and translation.

**Figure 3:**
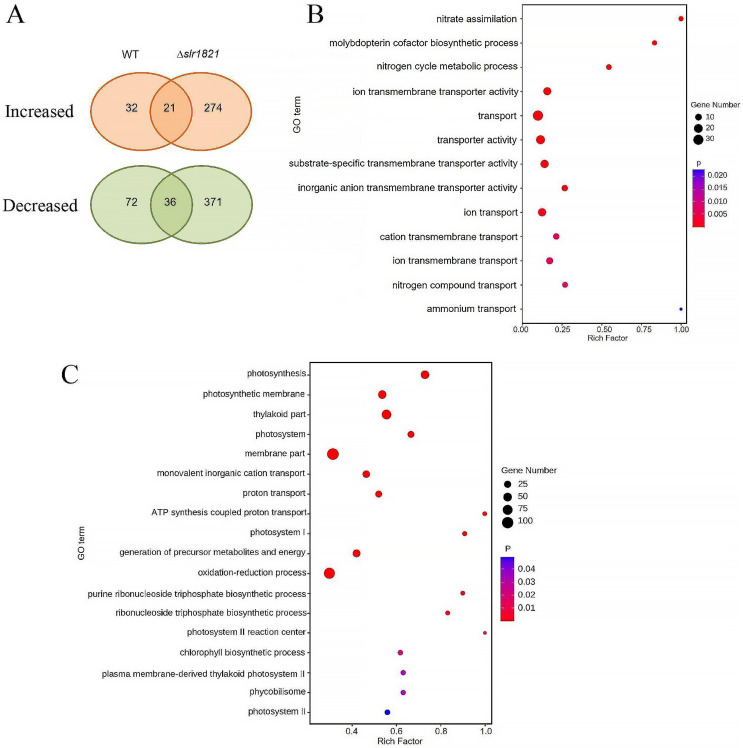
Transcriptional responses to NH_4_^+^ stress in ∆*slr1821* and the wild type (WT). (**A**) Total number of genes which were significantly increased or decreased in response to 30 min NH_4_^+^ stress in ∆*slr1821* and the WT. (**B**) DEGs-enriched representative GO categories (*p* < 0.05) in the WT upon NH_4_^+^ stress. (**C**) DEGs-enriched representative GO categories (*p* < 0.05) in ∆*slr182* upon NH_4_^+^ stress.

Secondly, to explore the primary NH_4_^+^-responsive genes in WT and reveal whether their regulation was dependent on the functional Slr1821, the transcriptomic responses of WT and ∆*slr1821* to NH_4_^+^ stress were compared (Figure 3). Fifty-three activated genes and 108 repressed genes were observed in WT, while the proper expression of 60% (32/53) of the NH_4_^+^-activated genes and 67% (72/108) of the NH_4_^+^-repressed genes were actually Slr1821-dependent since they were abolished or reversed in ∆*slr1821*. Thus, it supports the speculation that functional Slr1821 is essential for the regulation of early NH_4_^+^-responsive genes, both activated and repressed ones. In ∆*slr1821* upon NH_4_^+^ stress, the activated genes and repressed genes increased to 295 and 407, respectively. However, two large groups of DEGs, including 274 upregulated and 371 downregulated genes, were found only in NH_4_^+^-exposed ∆*slr1821* without responding to NH_4_^+^ in WT. They might be a secondary effect due to the loss of function of Slr1821. Comparison of the enriched GO categories of NH_4_^+^-responsive genes in WT and ∆*slr1821* revealed that “nitrate assimilation”, “molybdopterin cofactor biosynthetic process” and “ammonium transport” were among the most enriched biological processes of DEGs in the NH_4_^+^-exposed WT, while they were not observed in ∆*slr1821* (Figure 3B,C). The category “cation transport” was enriched in both WT and ∆*slr1821*. However, “photosynthesis”, “proton transport” and “ATP synthesis coupled proton transport” emerged as the most fluctuating biological process only in ∆*slr1821* but not in WT. The following enriched GO categories in ∆*slr1821* included “generation of precursor metabolites and energy”, “oxidation-reduction process”, “ribonucleoside triphosphate biosynthesis” and “chlorophyll biosynthesis”, all of which were not observed in WT. Such extensive fluctuation in photosynthesis and other metabolic pathways implied that ∆*slr1821* encountered severe NH_4_^+^ toxicity due to improper acclimation to NH_4_^+^ stress. On the other hand, “nitrate assimilation” and “ammonium transport” seemed to be the essential Slr1821-dependent processes for WT to acclimate to NH_4_^+^ stress.

### 2.3. Slr1821 Is Indispensable for the High Concentration NH4^+^-Induced Suppression of Nitrogen Uptake, Assimilation and Mobilization

**Figure 4:**
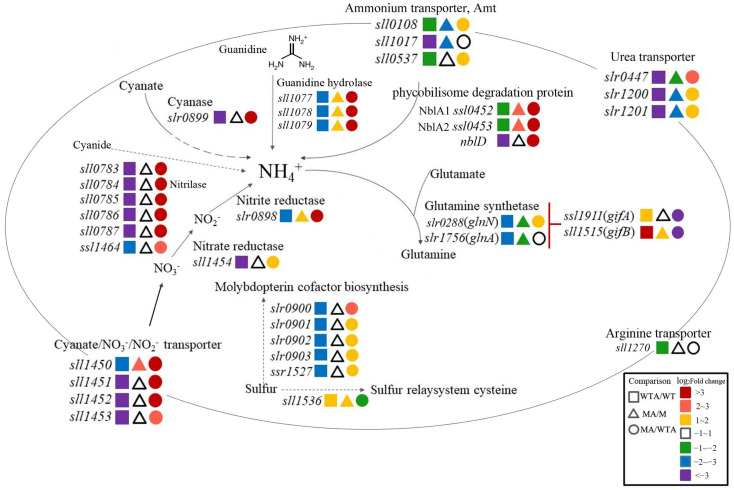
Comparison of transcript abundance of genes involved in nitrogen uptake and ammonium assimilation. Differential expressed key enzymes and transporters are included and presented as their gene symbol. The comparison pair and fold changes are indicated in different shapes and colors respectively. WT and WTA are wild type without and with NH_4_^+^ stress. M and MA are ∆*slr1821* knockout mutant without and with NH_4_^+^ stress.

First, we focus on how *Synechocystis* 6803 acclimates to high concentration of NH_4_^+^. Different from the redirection of sugar metabolism to storage reservoir upon nitrogen depletion [9], no significant change was observed in the central carbon metabolism-related genes in WT at 30 min upon NH_4_^+^ stress, such as glycolysis, TCA cycle, Calvin cycle and OPP pathway (Supplemental Table S2). However, a series of genes related with nitrogen uptake, mobilization and assimilation were notably suppressed in WT to avoid excess NH_4_^+^ toxicity, while such suppression was compromised or even reversed in ∆*slr1821*, suggesting the essential role of functional Slr1821 during the NH_4_^+^ stress acclimation (Figure 4).

#### 2.3.1. Nitrogen Uptake and Assimilation

Adjustment of nitrogen uptake and assimilation is critical for survival under fluctuating NH_4_^+^ availability. In contrast to the activation of nitrogen uptake and assimilation under nitrogen starvation [32], excess NH_4_^+^ notably suppressed genes related to nitrogen uptake and assimilation in WT (Figure 4). For example, genes encoding transport systems for ammonium (*amt1/sll0108*, *amt2*/*sll1017*, *amt3*/*sll0537*), urea (*urtABC*/*slr0447*, *slr1200*, *slr1201*), nitrate/nitrite (*nrtABCD/sll1450-53*), arginine and glutamine (*bgtB*/*sll1270*) were consistently suppressed two to twenty folds, thus implying the effective attenuation of excess nitrogen influx. Similar with the suppression of four nitrate/nitrite transporter subunits (*sll1450-53*), the downstream neighbor gene for ferredoxin-nitrate reductase (*sll1454*) was significantly repressed by NH_4_^+^ stress. Nitrate reductase and nitrite reductase catalyze successively to convert nitrate into NH_4_^+^. Reasonably, nitrite reductase-coding gene *slr0898* was also notably repressed by excess NH_4_^+^ (Figure 4).

Intriguingly, genes for the whole group of molybdopterin cofactor biosynthetic enzymes were coordinately repressed by excess NH_4_^+^, including *moeA/slr0900*, *moaA/slr0901*, *moaC/slr0902*, *moaE/slr0903* and *moaD/ssr1527*. They catalyzed the reaction from S-adenosyl-L-methionine to molybdopterin cofactor, the essential cofactor of nitrate reductase, while genes of another sulfur relay protein, *sll1536*, were upregulated, which might redirect excess sulfur from biosynthesis of molybdopterin cofactor to cysteine. Meanwhile, synergistically with the suppression of this group of genes, the upstream neighbor gene *slr0899* was also dramatically repressed. It encodes cyanase, catalyzing the reaction of cyanate with bicarbonate to produce ammonia and CO_2_, and its coding gene was reported previously to be transcribed as a part of a large transcription unit which was repressed by ammonium [33]. Its repression here could help to decrease the endogenous ammonium concentration upon NH_4_^+^ stress. The gene cluster *slr0898*-*slr0899-slr0900-slr0901-slr0902-ssr1527-slr0903* was reported previously in the same transcriptional unit [34]. Their synchronized suppression in WT was abolished congruously in ∆*slr1821*, suggesting that their co-suppression upon NH_4_^+^ stress was Slr1821-dependent.

#### 2.3.2. Ammonium Integration and Nitrogen Mobilization

Excess NH_4_^+^ also suppressed genes related to ammonium integration and mobilization of other nitrogen sources in WT (Figure 4). Endogenous NH_4_^+^ was assimilated into carbon compounds through several enzymatic reactions, among which glutamine synthetase (GS) catalyzing the ATP-dependent amidation of glutamate to form glutamine was the primary ammonium assimilation process. WT decreased the gene expression for GS at *slr0288* and *slr1756* to one sixth upon NH_4_^+^ stress, while it activated the expression for two GS-inactivating factors, IF7 (*ssl1911*) and IF17 (*sll1515*). Similar regulation was observed in ∆*slr1821* but to a lesser extent; for example, it decreased *slr0288* and *slr1756* only to half upon NH_4_^+^ stress, and it increased only one GS-inactivating factor, IF17 (*sll1515*).

Phycobilisomes are the photosynthetic light-harvesting complexes that store up to 50% of the cellular nitrogen and their ordered disassembly is part of the programmed response to nitrogen starvation; however, how phycobilisome degradation responds to excess NH_4_^+^ stress is not clear so far. Three small proteins, NblA1, NblA2 and NblD, are involved in the coordinated degradation of phycobilisome under nitrogen starvation [35,36,37]. Here, upon ammonium stress, their coding genes, *nblA1*, *nblA2* and *nblD*, were consistently repressed in WT (Figure 4). Interestingly, their suppression was all abolished in ∆*slr1821*; in contrast, increased expression was observed for NblA1 and NblA2 coding genes in ∆*slr1821*. Similarly, the ammonium repression of protease HtrA gene (*slr1204*) was absent in ∆*slr1821*.

Another two notable ammonium-suppressed gene clusters were associated with utilization of cyanide or guanidine as an alternative nitrogen source (Figure 4). One is the operon *sll0783-0784-0785-0786-0787-ssl1464*. The *sll0784* gene product is annotated as nitrilase, while the *sll0783* gene product was found to be essential for sustaining the reducing intracellular environment to accumulate polyhydroxybutyrate (PHB) upon nitrogen starvation [38]. Homologs of this conserved gene cluster are found to be essential for cyanide assimilation as a nitrogen source in nine different bacterial species [39]. The other operon involved comprised the guanidine hydrolase GdmH (Sll1077), two Ni^2+^-delivery proteins GhaA (Sll1078) and GhaB (Sll1079), and an ABC-type importer (Sll1080-1082). GdmH was recently discovered as a Ni^2+^-dependent guanidine hydrolase to break down guanidine into urea and ammonium, transcribed together with the Ni^2+^ chaperon GhaA (Sll1078) and GhaB (Sll1079) [40,41]. Here, the expression of both operons *sll0783-ssl1464* and *sll1077-1082* was significantly downregulated by excess NH_4_^+^, which might help alleviate the endogenous ammonium stress. It is reasonable to shut down cyanide assimilation, guanidine uptake and breakdown when ammonium is abundant as the preferred nitrogen source, but it is intriguing that such ammonium-induced suppressions were actually dependent on functional Slr1821. It was suggested that S1r1821 might act as an important point of sensing the excess NH_4_^+^, or as a negative response regulator to suppress expression of nitrogen uptake, assimilation and mobilization. Thus, we further analyzed the interference of Slr1821 knockout on the regulator and transporters.

### 2.4. Slr1821 Knockout Disrupts the Proper Response of Transporters and Channels in the Ammonium-Specific Stimulon

**Figure 5:**
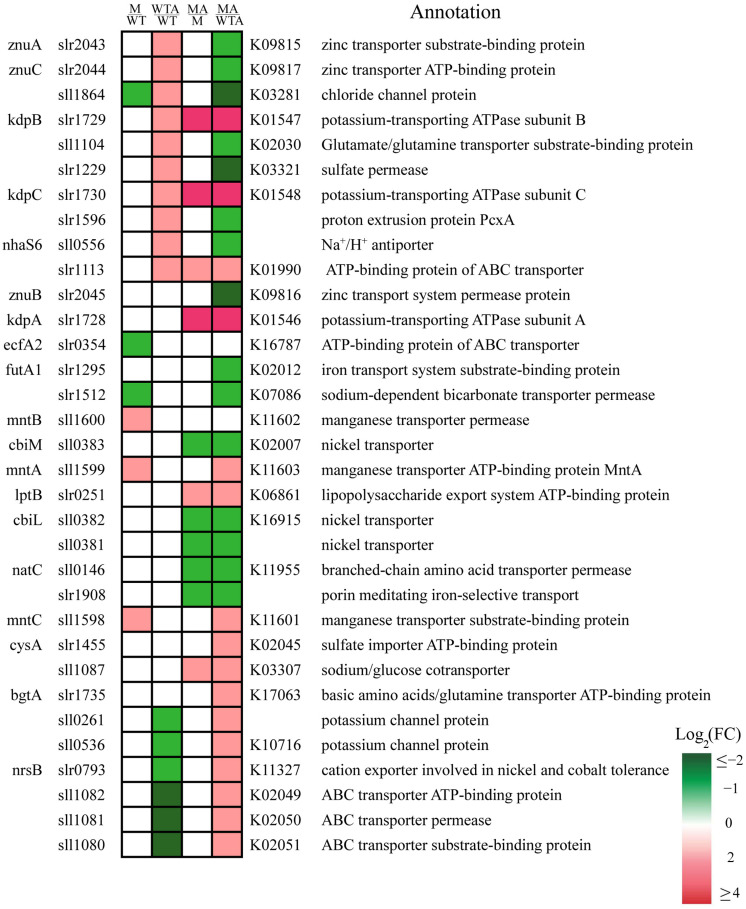
Ordered expression ranks for differentially expressed transporter-related genes. Ranks are ordered according to the log_2_(FC) of WTA/WT. The heatmap illustrates the log_2_(FC) of four comparisons in colors as indicated. WT and WTA are wild type without and with NH_4_^+^ stress. M and MA are ∆*slr1821* knockout mutants without and with NH_4_^+^ stress.

Clustering and functional enrichment analyses of DEGs clearly revealed that differential regulation of transporters and channels is an essential mechanism for excess NH_4_^+^ acclimation (Figure 5). As mentioned above, upon NH_4_^+^ stress WT attenuated the influx of nitrogen compound through suppression of transporters for ammonium, urea and nitrate/nitrite. Such attenuation somehow interferes with the general ion homeostasis including ion availability, which could be compensated or rescued by the regulation of specific transporters and channels in WT. However, knockout of Slr1821 obviously disturbed much of this regulation (Figure 5).

Firstly, WT increased the expression of anion transporters for chloride (*sll1864*) and sulfate (*slr1229*), cation transporters for zinc (*slr2043-2044*) and potassium (*slr1729-1730*), as well as proton exporter (*slr1596*), Na^+^/H^+^ antiporter (*sll0556*) and ABC transporters (*sll1104, slr1113*). Knockout of Slr1821 either eliminated the ammonium-induced activation or exaggerated the activation to a much higher level, thus rendering either much lower or much higher level of expression of these transporters in MA/WTA. Recently in shoots of *Arabidopsis*, it was reported that acidic stress resulting from excessive ammonium assimilation by plastidic glutamine synthetase caused ammonium toxicity [22]. We venture to speculate that *Synechocystis* 6803 has a similar mechanism. Then the activation of proton exporter PcxA (*slr1596*) might help alleviate the acidic stress in WT, while its diminished transcript abundance in ∆*slr1821* would exaggerate the ammonium toxicity.

Secondly, WT decreased the expression of potassium channel (*sll0536*), cation exporters of nickel and cobalt (*slr0793*) and an ABC transporter for guanidine (*sll1080-1082*). It was consistent with the repression of guanidine hydrolase as mentioned above (Figure 4). However, such ammonium-induced downregulation totally vanished in ∆*slr1821.*

Thirdly, other than the disrupted regulation on the ammonium-induced activation or suppression of transporters and channels, knockout of Slr1821 resulted in the transcriptional fluctuation of another group of transporters and channels. Higher expression in ∆*slr1821* compared with WT upon NH_4_^+^ stress (MA/WTA) was found in transporters for potassium (*slr1728*), manganese (*sll1558-1599*), basic amino acid (*slr1735*), lipopolysaccharide exporter (*slr0251*), sulfate importer (*slr1455*) and sodium/glucose cotransporter (*sll1087*). Lower expression (MA/WTA) was observed in transporters for iron (*slr1295*), zinc (*slr2045*), bicarbonate (*slr1512*), nickel (*sll0381-0383*), branched-chain amino acid (*sll0146*) and an iron-selective porin (*slr1908*). Transporter Slr1512 was involved in bicarbonate and pH-dependent acclimation mechanisms to high light stress [42]. NikKLMQO (*sll0381-sll0385*) was identified as nickel transporter, which is required for hydrogen production [43]. Slr1908 was recently reported to be primarily involved in inorganic iron uptake [44]. The ferric iron uptake protein FutA1 (Slr1295) was observed among the highest enhanced protein during salt stress, and has been suggested to be involved in protection of photosystem II under iron deficiency and in thylakoid membrane formation [45,46]. Therefore, downregulation of both *slr1908* and *slr1295* in ∆*slr1821* might hinder the availability of intracellular iron and interfere with the function of Fe-requiring protein, such as catalase, superoxide dismutase and cytochrome. In summary, the ion homeostasis including iron availability in ∆*slr1821* was actually destroyed and rendered the susceptibility to NH_4_^+^ stress.

### 2.5. Slr1821 Knockout Interferes with the Proper Response of Regulators upon Ammonium Stress

More than a dozen genes assigned regulatory functions responded to NH_4_^+^ stress in WT (Table 1), suggesting that extensive transcriptional reprogramming was required for the acclimation. Among the ammonium-activated regulators, the most impressive ones in WT were non-coding RNA Ncr1071 and transcriptional regulator Sll0998, both having about three folds upregulation. Ncr1071 was validated as the NtcA directly repressed the gene upon nitrogen limitation [32], while its target and function remained elusive. Sll0998 (RbcR) was recently reported as an important transcriptional activator for RuBisCo operon and genes related with carbon acquisition, thus positively regulating carbon assimilation [47]. However, how Sll0998 is regulated or controlled is still poorly understood. Here in our data, it is reasonable to find that *Synechocystis* upregulated RbcR expression during early acclimation to NH_4_^+^ stress, which might increase carbon assimilation to match the increase of nitrogen influx under high concentration of NH_4_^+^. Intriguingly, the ammonium-induced activation of both Ncr1071 and *sll0998* was eliminated in ∆*slr1821*, implying their activation upon NH_4_^+^ stress was dependent on functional Slr1821.

Ammonium also repressed expression of ten regulatory proteins (Table 1), among which three were previously reported to be activated by NtcA during early acclimation to nitrogen starvation [32], including the gene for nitrogen regulatory protein PII (*ssl0707*), the PII interactive protein PirC (*sll0944*) and two-component system response regulator Rre37 (*sll1330*). Interestingly, the ammonium-induced repression of PII was still retained in ∆*slr1821*, while ammonium-induced repression of PirC and Rre37 vanished, suggesting that functional Slr1821 was critical for ammonium regulation on PirC and Rre37, but not PII. As the pervasive signal processor, PII proteins decode intracellular metabolic status through binding key effectors such as ATP/ADP or 2-OG, and transduce these signals to various regulatory targets to orchestrate key steps of nitrogen and carbon metabolism, thus balancing the flow of metabolites for cell growth [10]. However, its function and regulation upon ammonium stress was not elucidated so far. Here, the ammonium-induced repression of PII provided a new viewpoint to study its function and regulation. PirC is the small interactive protein that switches binding between PII and the glycolytic enzyme PGAM (phosphoglycerate mutase), and PGAM is allosterically inhibited by PirC-PGAM interaction [9]. Here upon NH_4_^+^ stress, WT decreased expression of both PII and PirC (Table 1). It is tempting to speculate that WT relieved the PirC inhibition on PGAM, thereby allowing conversion of 3-phosphoglycerate (3-PGA) to 2-phosphoglycerate (2-PGA) through PGAM, which is the key step to remove 3-PGA (the first stable product of CO2 fixation) from the Calvin–Benson–Bassham (CBB) cycle towards the lower part of glycolysis, thus supplying carbon skeleton for cell growth. Here, the increase of carbon skeleton to match the excess ammonium supply seems logical. In contrast, ∆*slr1821* decreased expression of only PII but not PirC, which might exacerbate inhibition of PirC on PGAM, thus hindering the carbon flow from CBB to glycolysis and the downstream anabolic metabolism for cell growth. It could partially contribute to the ammonium intolerance of ∆*slr1821.*

Consistent with the activated carbon assimilation suggested by upregulation of Sll0998, several regulators on sugar catabolism were repressed in WT, such as Rre37 (*sll1330*) and PrqR (*slr0895*). Rre37 was indispensable for the light-activated heterotrophic growth through controlling the expression of glycolytic genes [48]; while under nitrogen starvation, Rre37 stimulated accumulation of glycogen and TCA cycle metabolites, including 2-oxoglutarate [49]. PrqR was previously suggested to regulate glucose metabolism and oxidative stress acclimation [50]. Ammonium-induced repression was also observed in two-component protein kinase Sll0782, response regulator Slr1214 and group 3 sigma factor SigG (*slr1545*) (Table 1). Gene *slr1214* were reported previously to be induced by acid stress and inorganic carbon limitation [51,52], and it was also part of a UV-A-activated signaling system that is required for negative phototaxis [53]. Downregulation of SigG was reported during early acid acclimation [30], as well as transiently under salt, heat and high-light stress [54]. Among the ten ammonium-repressed regulators, three (Sll1293, Sll1294 and Sll1296) were suggested to be involved in chemotaxis signal transduction. Conserved motifs in Sll1293, Sll1294 and Sll1296 implied that they were the two-component signaling systems regulating bacterial chemotaxis.

Knockout of the *slr1821* gene not only abolished more than 50% of the ammonium- induced or -repressed expression of regulators as mentioned above but also resulted in abnormal regulation of other regulators (Table 1). For example, ammonium-induced activation of Sll0528, SigD, two component sensor histidine kinase Sll1590 and response regulator Sll1592 and PerR, and ammonium-induced suppression of global nitrogen regulator NtcA, transcription regulator CyAbrB2 and histidine kinase Slr0473 were exclusively observed in ∆*slr1821*. As the S2P homolog, Sll0528 was implicated in multiple stress responses, such as salt, cold and acid [31,55,56]. PerR (Slr1738) controlled the defense against metal and oxidative stress [57,58]. The SigD factor was upregulated in high light, heat, singlet oxygen and H_2_O_2_ stresses, and was regarded as central regulator of oxidative stress acclimation [59,60]. SigD was also suggested to regulate the partitioning of carbon skeletons in low nitrogen, since an abnormally high glycogen content was detected in Delta sigBCE strain (SigD is active) [61]. Here in ∆*slr1821*, simultaneous upregulation of *sll0528, perR* and *sigD* might indicate that aggravated oxidative stress resulted from improper acclimation to ammonium. The ammonium-induced activation of *sll0528* could not rescue the susceptibility of ∆*slr1821* to a high concentration of ammonium, indicating Sll0528 could not functionally compensate the loss of Slr1821, though they were S2P homologs.

NtcA is recognized as the global nitrogen regulator to directly activate at least 51 genes and repress 28 genes during early acclimation to nitrogen starvation in *Synechocystis* 6803 [32]. Since this NtcA regulon represented the majority of early nitrogen starvation-responsive genes and some of them were found reversely regulated to ammonium stress, thus their response to ammonium were analyzed in detail here. Seventeen out of forty-six detected NtcA-dependent N-starvation-activated genes were repressed upon NH_4_^+^ stress in WT, accounting for 16% of the ammonium-repressed genes, while only four out of twenty-four detected NtcA-directly repressed genes were activated, accounting for 8% of the ammonium-activated genes. Therefore, although NtcA regulation was the center of early acclimation to nitrogen starvation, it was only part of the story during early acclimation to NH_4_^+^ stress. Obviously, acclimation to a high concentration of NH_4_^+^ is not merely the reverse response to nitrogen starvation, partly due to the ammonium toxicity. Intriguingly, NtcA transcript abundance did not change in WT upon ammonium stress; however, it was significantly repressed in *△slr1821* upon NH_4_^+^ stress. Such repression might be the compensation of NH_4_^+^ stress inadaptability due to knockout of *slr1821*. Similar compensation was also observed in the transcriptional regulator CyAbrB2 gene *sll0822*. CyAbrB2 was involved in coordination of carbon and nitrogen metabolism, essential for the gene’s activation upon a shift to nitrogen starvation or photomixotrophic conditions; thus, it was suggested to work in parallel with NtcA to achieve flexible regulation of the nitrogen uptake system [62,63,64].

Opposite regulation on genes for assimilation of carbon and nitrogen, such as repression of nitrogen regulatory protein PII, PII interactive protein PirC and activation of carbon acquisition regulator RcbR, demonstrated a striking coordination of regulation to balance C- and N-fluxes in WT upon NH_4_^+^ stress. Slr1821 was critical for part of this carbon/nitrogen homeostasis control.

### 2.6. Knockout of slr1821 Resulted in Defective Photosynthesis, Compromised Translational and Transcriptional Machinery and Redirection of Carbon Flux

#### 2.6.1. Defective Photosystem and Carbon Fixation

Knockout of *slr1821* resulted in extensive repression of photosynthesis and carbon fixation upon NH_4_^+^ stress, such as 65%, 73%, 25% and 33% genes significantly downregulated in photosynthesis, antenna proteins, porphyrin and chlorophyll biosynthesis, and carbon fixation, respectively (Supplemental Tables S3–S6). In ∆*slr1821* upon NH_4_^+^ stress, consistent with the suppression in photosynthesis apparatus and accelerated degradation of phycobilisome as mentioned above, most genes of the critical enzymes in Calvin cycle were significantly repressed, such as genes for the committed enzyme Ribulose-bisphosphate carboxylase (Rubisco) large and small subunit (*slr0009* and *slr0012*), phospho-glycerate kinase (*slr0394*), triose phosphate isomerase (*slr0783*), sedoheptulose bisphosphatase (*slr2094*), transketolase (*sll1070*), phosphopentose epimerase (*sll0807*) and phosphoribulose kinase (*sll1525*). While WT only notably repressed 11% of photosynthesis genes, it had no significant regulation on genes related with antenna proteins, porphyrin and chlorophyll biosynthesis and carbon fixation. Thus, it was confirmed that with functional Slr1821, WT successfully acclimated to high concentration of NH_4_^+^.

**Figure 6:**
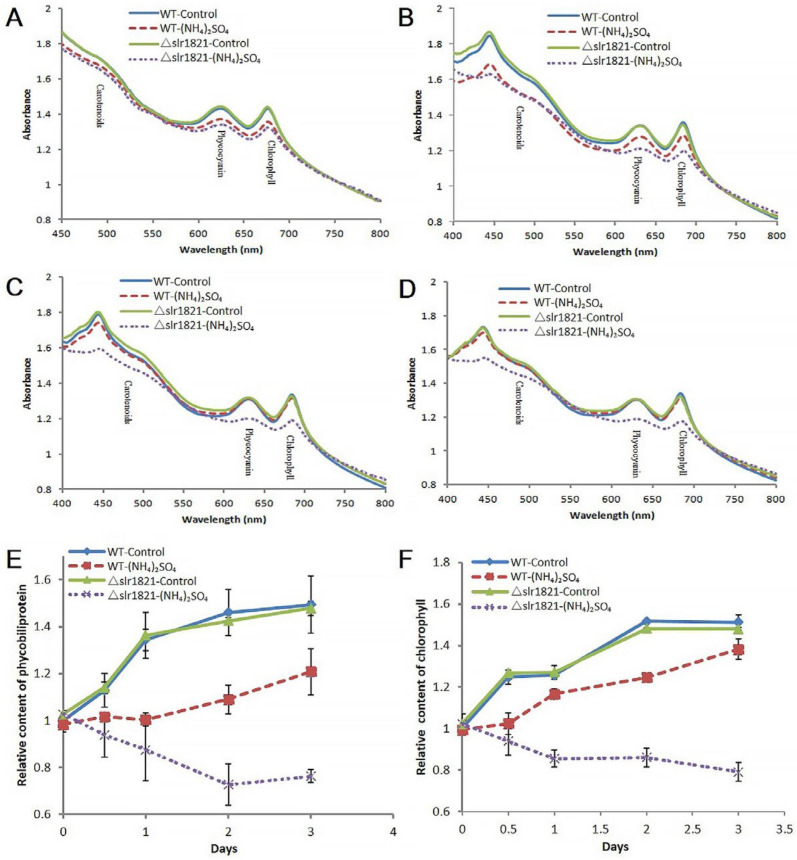
Defective photosystem in ∆*slr1821* upon NH_4_^+^ stress. (A)-(D). The whole cell absorption spectra of WT and ∆*slr1821* with or without 15 mM (NH_4_)_2_SO_4_ at the first (**A**), second (**B**), third (**C**) and fourth (**D**) days, respectively. (**E**) The relative content of phycobiliprotein in WT and ∆*slr1821* with or without 15 mM (NH_4_)_2_SO_4_ during the first three days. Relative content was data normalized to WT control in day 0 setting as 1. (**F**) The relative content of chlorophyll in WT and ∆*slr1821* with or without 15 mM (NH_4_)_2_SO_4_ during the first three days. Relative content was data normalized to WT control in day 0 setting as 1.

Defective photosystem was confirmed through the physiological characterization (Figure 6). The whole cell absorption during the first four days indicated that WT recovered from NH_4_^+^ toxicity at the third day, while ∆*slr1821* could not recover and had significantly less chlorophyll, phycocyanin, phycobiliprotein and carotenoids.

#### 2.6.2. Disrupted Oxidative Phosphorylation and Central Carbon Metabolism

Knockout of *slr1821* resulted in extensive repression in 35% genes of oxidative phosphorylation upon NH_4_^+^ stress, including subunits for NADH dehydrogenase, cytochrome c oxidase and ATP synthase (Supplemental Table S7), thus rendering the defective electron transfer and oxidative phosphorylation to provide energy for cellular processes.

The glycolysis (Embden–Meyerhof–Parnas (EMP) pathway), oxidative pentose phosphate (OPP) pathway and Entner–Doudoroff (ED) pathway are the three glucose degradation pathways essential for *Synechocystis* carbon metabolism [65]. In contrast to the mainly unchanged carbon metabolism in WT upon ammonium stress, knockout of *slr1821* resulted in repressed glycolysis (EMP) pathway and the ED pathway, as well as disruption of the OPP pathway (Supplemental Table S2). Suppression of the EMP pathway and ED pathway was indicated by the downregulation of genes for enzymes catalyzing the three committed steps in the EMP pathway, glucokinase (*sll0593*), phosphofructokinase (*sll1196*) and pyruvate dehydrogenase subunit (*slr1934*), as well as the downregulation of the gene for the ED pathway enzyme phosphogluconate dehydratase (*slr0452*). Disruption of the OPP pathway was implied as the upregulation of the gene for 6-phosphogluconate dehydrogenase (*sll0329*) and the downregulation of the gene for transketolase (*sll1070*).

Knock-out of *slr1821* did not change gene expression of the TCA cycle remarkably; and expression of most TCA genes remained constant upon NH_4_^+^ stress in both WT and ∆*slr1821*, except *sll1981*. It was significantly upregulated in ∆*slr1821* upon NH_4_^+^ stress. It encoded the alpha-ketoglutarate decarboxylase, catalyzing conversion of alpha-ketoglutarate (2-OG) to succinic semialdehyde in the special TCA cycle of *Synechocystis* 6803 [66,67]. Since 2-OG accumulation usually indicates the higher carbon/nitrogen ratio, upregulation of its conversion here might imply the abnormal 2-OG level due to knock-out of *slr1821*.

#### 2.6.3. Compromised Translational and Transcriptional Machinery

Among the 50 genes encoding ribosomal proteins, 34 (68%) were significantly repressed in ∆*slr1821* compared with WT without NH_4_^+^ stress (M/WT) (Supplemental Table S8), which might interfere with the translation efficiency in mutants since ribosomal proteins are the structural and functional foundation of the translational apparatus-ribosome. Upon NH_4_^+^ stress, WT did not suppress gene expression of ribosomal protein, but activate two of them, the 50S ribosomal protein L9 and L13; while ∆*slr1821* suppressed 19 ribosomal protein genes. Thus, the repression in ∆*slr1821* compared with WT upon NH_4_^+^ stress (MA/WTA) was even more aggravated, as 92% (46/50) of the ribosomal protein-coding genes were notably repressed (Supplemental Figure S3A). Meanwhile, upon NH_4_^+^ stress, ∆*slr1821* suppressed several aminoacyl-tRNA synthetase genes (Supplemental Table S9), such as subunits for glutamyl-tRNA amidotransferase and phenylalanine–tRNA ligase, which catalyzed the attachment of specific amino acid to its corresponding tRNA during protein translation. Thus, it resulted in the downregulation of 54% (14/26) of genes for aminoacyl-tRNA synthetase (Supplemental Figure S3B). In ∆*slr1821*, compromised expression of ribosomal apparatus and amino acid-tRNA ligase implied the severely damaged translational machinery, which could not efficiently synthesize the stress-responsive proteins or repair the stress-destroyed proteins. Consistent with the suppression on translational machinery, Slr1821 knockout also diminished the expression of RNA polymerase; one and three genes for RNA polymerase subunits were downregulated with and without NH_4_^+^ stress, respectively. Particularly, gene *sll1818* for RNA polymerase alpha subunit was repressed even without NH_4_^+^ stress, thus implying that the Slr1821 knockout might impair both transcriptional and translational machinery.

#### 2.6.4. Interfered Direction of Carbon Flux among PHB and Other Carbon Reservoirs

*Synechocystis* 6803 produces glycogen, fatty acid and polyhydroxybutyrate (PHB) as energy and carbon reserves to combat adverse conditions, such as nitrogen starvation or phosphorus depletion. PHB represents a promising bioplastic alternative with good biodegradation properties, thus its higher accumulation is the target of biotechnology applications [68]. Here intriguingly, knockout of *slr1821* resulted in notable upregulation of genes for three catalytic enzymes to produce PHB from acetyl-CoA, including PhaA/*slr1993*, PhaB/*slr1994* and PhaE/*slr1829*, as well as the activation of genes for glycogen-debranching enzyme (*slr1857*) and glycogen phosphorylase (*slr1367*), which decompose glycogen to generate acetyl-CoA as a precursor for PHB synthesis (Supplemental Figure S4). In addition, gene *ssl2501* encoding PHA surface-coating protein (phasin), PhaP, was also upregulated due to the loss of *slr1821*. PhaP was reported to contribute to the biosynthetic activity of PHB synthase and regulate the PHB granule formation [69]. Therefore, it was suggested that knockout of *slr1821* influenced the carbon homeostasis and might redirect the carbon flux to PHB. Upon ammonium stress, the unusual upregulation of these PHB synthesis-related genes due to *slr1821* knock-out still persisted or was even more pronounced; and additionally, the *slr0058-slr0061* operon was unexpectedly upregulated in mutant. The Slr0058 protein was reported as a novel regulatory protein involved in PHB granule formation [70]. Deletion of *slr1821* seemed to enhance the whole PHB biosynthesis process and the enhancement was even more prominent under ammonium stress. However, whether the PHB amount was enhanced in mutant awaits further investigation since compromised photosynthesis might hinder the carbon source. Meanwhile, in wild type, the operon *sll0783-ssl1464* was notably repressed upon ammonium stress, and such ammonium-induced repression was completely absent due to the *slr1821* knock-out. The nitrogen starvation-induced protein Sll0783 is required to maintain the reducing state, for example, NADPH pool for PHB accumulation [38,71]. Here, it was intriguing to observe its ammonium-induced repression was dependent on the functional Slr1821. Since PHB production is the redirection of carbon and energy upon limited nutritional supply with excess carbon source, the unexpected activation of PHB biosynthesis- related genes in mutant implied that functional Slr1821 is essential to monitor or maintain the carbon/nitrogen hemostasis.

Coordinately, genes related with biosynthesis of another carbon and energy reservoir, fatty acid, were found reduced in ∆*slr1821*. For example, genes *sll1655* and *sll0053* for acetyl-CoA carboxylase, *sll1069* for beta-ketoacyl synthase and *slr0886* for beta-ketocayl-ACP reductase were all downregulated in ∆*slr1821* upon NH_4_^+^ stress, while no notable change was observed in WT. Intriguingly, biosynthesis of another carbon storage polymer, glycogen, might also be downregulated, since gene *sll0726* for phosphoglucomutase and *slr1176* for glucose-1-phosphate adenylyltransferase were both significantly downregulated in ∆*slr1821* upon NH_4_^+^ stress, while no notable change was observed in WT. Such abnormal redirection of carbon together implied the critical role of Slr1821 in controlling the homeostasis of carbon/nitrogen under fluctuating nitrogen conditions.

#### 2.6.5. Extensive Stress Response

In addition to the notable upregulation of general stress regulators as mentioned above, ∆*slr1821* increased expression of a series of stress-responsive genes upon NH_4_^+^ stress, such as *sodB* (*slr1516*) encoding superoxide dismutase, GST (Glutathione-S-Transferase) gene *sll1545*, *slr0851* (*ndbA*) for type II NADH dehydrogenase, *sll1514* for heat shock protein HspA, *sll1867* for photosystem II D1 protein and *slr1641* coding chaperone protein. Many other ammonium-stimulated genes in ∆*slr1821* have no functional annotations, however SodB, NdbA, GST, HspA, D1 were all implicated in multiple stress responses of *Synechocystis* 6803 [11,72,73,74,75]. Thus, the pronounced activation of these stress- responsive genes implied more severe stress experienced in ∆*slr1821* upon NH_4_^+^ stress.

## 3. Materials and Methods

### 3.1. Strains and Cultivation

Derived from ATCC27184, *Synechocystis* 6803 was cultivated in BG11 medium supplemented with 20 mM HEPES-NaOH (pH 7.5) at continuous illumination (~30 μE·m^−2^·s^−1^) and 29 °C. The ∆*slr1821* strain was maintained with kanamycin (50 μg mL^−1^). For ammonium stress, cultures were cultivated in BG11 medium supplemented with 15 mM (NH_4_)_2_SO_4_.

### 3.2. Construction of ∆slr1821 Strain

The *slr1821* upstream region was obtained by PCR amplification of chromosomal DNA using forward primer 5′-CGCGGATCCAGCATTAACCT-CGCTGTG-3′ and reverse primer 5′-CCGGAATTCGTAGGGTCAATAACTCTTCCA-3′. It was ligated with *Hind III*/*Sal I* digested pUC118 to generate the plasmid pUC118-Re (Supplemental Figure S1). The *slr1821* downstream region was obtained using forward primer 5′-CCCAAGCTTGTCTAAGCCAGCAATCCA-3′ and reverse primer 5′-ACGCGTCGACCAAATCCTAAGGCAAAGC-3′. It was ligated with *BamH I*/*EcoR I* digested pUC118-Re to generate the plasmid pUC118-Re-Le. The kanamycin resistance gene (Km^r^) from pET-30b was ligated with *Sal I*/*BamH I* digested pUC118-Re-Le to generate pUC118-Re-Le-Km^r^, which was subsequently transformed into *Synechocystis* 6803 wild type through homologous recombination. Full segregation of ∆*slr1821* was achieved after extensive cultivation under rising kanamycin antibiotic pressure and confirmed by PCR and sequencing.

### 3.3. RNA Extraction, Library Construction, Sequencing and Bioinformatics Analysis

Total RNA was extracted from tissue samples using TRIzol^®^ Reagent (Invitrogen, Carlsbad, CA, USA) according to the manufacturer’s instructions and genomic DNA was removed using DNase I (TaKara, Osaka, Japan). The RNA quality was determined by 2100 Bioanalyser (Agilent, Santa Clara, CA, USA) and quantified using the ND-2000 (NanoDrop Technologies, Waltham, MA, USA). Only high-quality RNA sample (OD260/280 = 1.8~2.0, OD260/230 ≥ 2.0, RIN ≥ 6.5, 28S:18S ≥ 1.0, ≥100 ng/μL, ≥2 μg) was used to construct the sequencing library. The RNA-sequencing library was prepared following TruSeq^TM^ RNA sample preparation Kit from Illumina (San Diego, CA, USA) using 2 μg of total RNA. Firstly, ribosomal RNA (rRNA) was depleted by Ribo-Zero Magnetic kit (Epicenter) and total mRNAs were broken into short fragments of 200 nt by adding a fragmentation buffer. Then double-stranded cDNA was synthesized using SuperScript double-stranded cDNA synthesis kit (Invitrogen, Carlsbad, CA, USA) with random hexamer primers (Illumina, San Diego, CA, USA). When the second strand cDNA was synthesized, dUTP was incorporated in place of dTTP. The synthesized cDNA was subjected to end-repair, phosphorylation and adaptor addition. The second strand cDNA with dUTP was recognized and degraded by UNGase. Libraries were size-selected for cDNA target fragments of 200 bp on 2% Low Range Ultra Agarose and followed by PCR amplification using Phusion DNA polymerase (Finnzymes, Espoo, Finland) for 15 cycles. After quantification by TBS380, the paired-end RNA sequencing library was sequenced with Illumina HiSeq×TEN in 2 × 150 bp read length.

The processing of original images to sequences, base-calling and quality value calculations were performed using the Illumina GA Pipeline (version 1.6), in which 150bp paired-end reads were obtained. Clean reads were selected by removing low-quality sequences, reads with more than 5% of N bases (unknown bases) and adaptor sequences. Clean reads in each sample were mapped to the reference genome by Bowtie2 (http://bowtie-bio.sourceforge.net/bowtie2/index.shtml, accessed on 30 March 2023). Expression level of each gene was calculated in FPKM to represent fragments per kilobase of exon model per million mapped reads using RSEM (http://deweylab.github.io/RSEM/, accessed on 30 March 2023). Differential expression analysis among groups was performed based on FPKM using edgeR (http://www.bioconductor.org/packages/2.12/bioc/html/edgeR.html, accessed on 30 March 2023). The GO term covering cellular component, molecular function and biological process was assigned to each gene (Gene Ontology, http://www.geneontology.org/, accessed on 30 March 2023). The GO terms statistically significantly enriched with differentially expressed genes (DEGs) were identified using Goatools (https://github.com/tanghaibao/GOatools, accessed on 30 March 2023) to illustrate the difference between two particular groups on functional levels. After multiple corrections including Bonferroni, Holm and Sidak, GO terms with adjusted *p*-value ≤ 0.05 were recognized as significantly enriched with DEGs. After assigning the KEGG annotation to genes, the KEGG term enriched with DEGs was identified through KOBAS 2.0 (http://kobas.cbi.pku.edu.cn, accessed on 30 March 2023). Similar with the GO enrichment analysis, after multiple corrections including Bonferroni, Holm and Sidak, KEGG terms with adjusted *p*-value ≤ 0.05 were recognized as significantly enriched with DEGs to indicate the most-affected metabolic pathways or signal transduction pathways that the DEGs were involved in.

### 3.4. Physiological Characterization

The growth curve and whole cell absorbance was measured by UV2300 spectrophotometer (Techcomp, Beijing, China). The extraction and quantification for chlorophyll and PBP content were performed as described previously [30]. Data are presented as mean ± standard deviation (SD) of three independent biological replicates. Statistical comparisons were performed by one-way analysis of variance (ANOVA) followed by hypergeometric test (R language). A *p*-value < 0.05 was considered to be statistically significant.

### 3.5. RNA Isolation and Quantitative RT-PCR

Total RNA was extracted using the bacterial total RNA extraction kit (DongSheng Biotech, Guangzhou, China) according to the manufacturer’s instruction. The DNA residue was removed by DNase and DNA contamination was examined by PCR amplification without reverse transcription. Quantitative RT-PCR was performed using one-step SYBR Green I kit (TAKARA Biotech, Dalian, China) on ABI7500 (Life Technologies, Grand Island, NY, USA) with the following conditions: reverse transcription program as 42 °C for 5 min, 95 °C for 10 s; amplification and quantification program repeated for 40 cycles of 95 °C for 5 s, 60 °C for 34 s; melting curve program as 95 °C for 15 s, 60 °C for 1 min and 95 °C for 15 s. The *rnpB* gene that encodes RNA subunit of ribonuclease P was used as an internal control. The primers sets are listed in Supplemental Table S10. Melting curve analysis was performed after each run to confirm the homogeneity in the amplification. Three independent biological replicates for each sample and three technical replicates of each biological replicate were analyzed. For the reactions, a master mix of the following components was prepared: 10 μL 2× One step SYBR RT-PCR Buffer, 3.2 μL RNase-Free H_2_O, 0.8 μL (0.4 μM) forward primer, 0.8 μL (0.4 μM) reverse primer, 0.8 μL PrimeScript one-step enzyme mix, 0.4 μL ROX and 4 μL total RNA. The relative expression of genes was calculated using the 2^−ΔΔ*C*t^ method.

## 4. Conclusions

Through physiological characterization and transcriptomic analysis of Slr1821 knockout mutant, the S2P homolog Slr1821 is found essential to the effective acclimation of *Synechocystis* 6803 to high concentrations of NH_4_^+^. The proper expression of 60% (32/53) of the NH_4_^+^-activated genes and 67% (72/108) of the NH_4_^+^-repressed genes were actually Slr1821-dependent since they were abolished or reversed in ∆*slr1821*. *Synechocystis* 6803 suppressed nitrogen uptake and assimilation, ammonium integration and mobilization of other nitrogen source, such as phycobilisome degradation, upon NH_4_^+^ stress. Opposite regulation on genes for assimilation of nitrogen and carbon, such as repression of nitrogen regulatory protein PII, PII interactive protein PirC and activation of carbon acquisition regulator RcbR, demonstrated that *Synechocystis* 6803 coordinated regulation to maintain carbon/nitrogen homeostasis under increasing nitrogen, while functional Slr1821 was indispensable for most of this coordinated regulation. Additionally, *slr1821* knockout disrupted the proper response of regulators and transporters in the ammonium-specific stimulon and resulted in defective photosynthesis. Transcriptomic data also suggested compromised translational and transcriptional machinery, disrupted oxidative phosphorylation and central carbon metabolism, as well as redirection of carbon flux as the consequence of loss of functional Slr1821. For the first time, ammonium stress acclimation was investigated in detail in cyanobacteria, and S2P homolog Slr1821 was revealed to be the critical point controlling the C/N homeostasis during this acclimation. The results provide new insight into the coordinated regulation to maintain C/N homeostasis during nutritional fluctuations and the functional characterization of photosynthetic S2P homologs. They also provide a foundation for bioremediation of high-ammonium-containing wastewater using cyanobacteria and give hints to improve the ammonium tolerance in crop plants.

## Figures and Tables

**Table 1:** Differential expression of regulatory protein or element.

Gene	Annotation of Gene Product	Log_2_FC			
		M WT	WTA WT	MA M	MA WTA
*ncr1071*	non-coding RNA, Ncr1071	−2.25	1.76	0.52	−3.48
*sll0998*	LysR-type transcriptional regulator, RbcR	−0.89	1.4	−0.87	−3.16
*slr1731*	sensor histidine kinase, KdpD	0.67	1.1	5.36	4.93
*sll0528*	zinc metalloprotease	0.43	0.74	3.73	3.42
*sll1592*	two-component response regulator	0.35	0.33	2.17	2.19
*sll0822*	transcriptional regulator cyAbrB2	−0.61	0.17	−2.64	−3.42
*sll1590*	two-component sensor histidine kinase	0.16	0.14	3.11	3.13
*sll1423*	global nitrogen regulator, NtcA	−0.57	0.01	−1.29	−1.86
*slr1738*	transcription regulator PerR	1.21	−0.08	1.13	2.43
*slr0473*	cyanobacterial phytochrome 1, two-component sensor histidine kinase	−0.16	−0.33	−1.39	−1.22
*sll2012*	group 2 RNA polymerase sigma factor, SigD	−0.08	−0.49	1.44	1.84
*slr0895*	transcriptional regulator, PrqR	−0.37	−1.07	−2.04	−1.35
*sll1293*	chemotaxis signal transducer	0.06	−1.19	−0.2	1.05
*slr1545*	group 3 RNA polymerase sigma factor, SigG	0.63	−1.34	−1.7	0.27
*sll1330*	two-component system response regulator, Rre37	0.38	−1.44	0.2	2.02
*sll0782*	protein kinase, transcriptional regulator	0.77	−1.58	0.8	3.16
*slr1214*	two-component response regulator PatA subfamily	0.55	−1.66	−1.03	1.17
*sll1294*	methyl-accepting chemotaxis protein	−0.3	−1.88	−0.6	0.98
*sll1296*	two-component hybrid sensor and regulator	−0.76	−2.15	−1.29	0.11
*sll0944*	PII interactive protein, PirC	0.67	−2.28	−0.61	2.34
*ssl0707*	nitrogen regulatory protein PII	−0.19	−2.64	−2.56	−0.11

Ranks are ordered according to the log_2_FC of WTA/WT. WT and WTA are wild type without and with NH_4_^+^ stress. M and MA are ∆*slr1821* knockout mutant without and with NH_4_^+^ stress.

## Data Availability

The RNA-sequencing data have been deposited in NCBI’s Sequence Read Archive (SRA) and accession numbers will be provided during review.

## Supplementary Materials

The supporting information can be downloaded at: https://www.mdpi.com/article/10.3390/ijms24076606/s1.
