# Supplementary material for: Site-2 Protease Slr1821 Regulates Carbon/Nitrogen Homeostasis during Ammonium Stress Acclimation in Cyanobacterium Synechocystis sp. PCC 6803
Source: Int J Mol Sci. 2023 Apr 1;24(7):6606. doi: 10.3390/ijms24076606 (PMC10094980; doi:10.3390/ijms24076606)
Supplement: Supplementary file 1 [file ijms-24-06606-s001.zip › Supplementary Figures and Tables.pdf]

**Supplemental Table S1. Statistics result of RNA sequencing data.**

| Sample_ ID | Raw Data Total_Reads | Raw Data Total_Bases | Clean Data Total_Reads | Clean Data Total_Bases | Error% | Q20% | Q30% | GC% |
|---|---|---|---|---|---|---|---|---|
| M-1 | 19019678 | 2.87E+09 | 18885526 | 2.73E+09 | 0.0109 | 98.78 | 96.62 | 49.23 |
| M-2 | 20045072 | 3.03E+09 | 19596762 | 2.81E+09 | 0.0119 | 98.43 | 95.42 | 49.15 |
| M-3 | 20440760 | 3.09E+09 | 20012366 | 2.86E+09 | 0.0118 | 98.48 | 95.55 | 49.49 |
| MA-1 | 24578712 | 3.71E+09 | 24028760 | 3.45E+09 | 0.0119 | 98.41 | 95.38 | 49.12 |
| MA-2 | 22051244 | 3.33E+09 | 21507562 | 3.08E+09 | 0.0122 | 98.3 | 95.11 | 49.01 |
| MA-3 | 23445508 | 3.54E+09 | 22897854 | 3.28E+09 | 0.012 | 98.37 | 95.29 | 49.35 |
| WT-1 | 15365482 | 2.32E+09 | 15167632 | 2.13E+09 | 0.0106 | 99.05 | 96.98 | 52.42 |
| WT-2 | 20409760 | 3.08E+09 | 20153882 | 2.85E+09 | 0.0106 | 99.06 | 97 | 51.36 |
| WT-3 | 19982788 | 3.02E+09 | 19445352 | 2.78E+09 | 0.0124 | 98.22 | 94.86 | 51 |
| WTA-1 | 24030246 | 3.63E+09 | 23478232 | 3.37E+09 | 0.0121 | 98.36 | 95.23 | 50.85 |
| WTA-2 | 24443248 | 3.69E+09 | 23900728 | 3.43E+09 | 0.012 | 98.4 | 95.31 | 51.26 |
| WTA-3 | 24981840 | 3.77E+09 | 24438662 | 3.51E+09 | 0.0119 | 98.42 | 95.37 | 50.78 |

**Supplemental Table S10. Sequences of primers using in RT-qPCR.**

| Name | Sequences of primers |
|---|---|
| rnpB-S | GAGTTAGGGAGGGAGTTGC |
| rnpB-A | GTGCAGGATGACGGAGAAA |
| sll1017-S | TGCTGCGGACAGAGTGGT |
| sll1017-A | GTGGGCTGGTATGGCTTT |
| slr1201-S | AAGTCACCGTCCTGGCTCT |
| slr1201-A | CCCTGGTTTCATCGTCCC |
| slr0901-S | TTATTTCTGCCCAAGAGT |
| slr0901-A | ACCGATTATGCCTCCACA |
| sll0783-S | TAACACCGGGACCATAGA |
| sll0783-A | TACAGTGGCGTTTGAAGG |
| sll1327-S | GAGCCACTAGGGAATCGG |
| sll1327-A | GGCAGTAAAGCCAAGCAAT |
| slr1291-S | ACCTACGACCGTACCCAC |
| slr1291-A | AAAGAAGCCATAGCACCC |
| slr0851-S | CGGTGTTGACTGAAAGAATG |
| slr0851-A | GAGGCTAGAAGCCCTGAC |
| slr0952-S | ATATCCGCATTCCCAACC |
| slr0952-A | CGGGCAGTGTAACCTTCG |
| sll1590-S | GTGTCATTGCGGTAAACATC |
| sll1590-A | CCCTTAATGCCTTCCAAGC |
| slr1728-S | AGAAGTGCGCTTTGGTTG |
| slr1728-A | GGAATAGGTCCGATAGGGT |

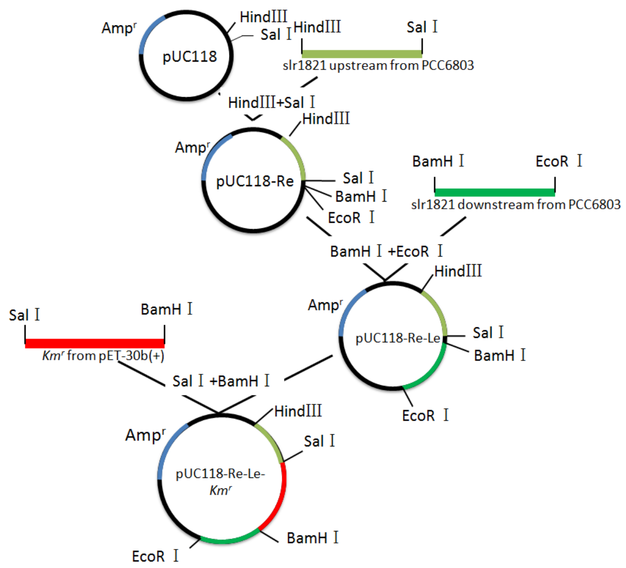


**Supplemental Figure S1. Construction of the plasmid for homologous recombination to knockout *slr1821* gene.**

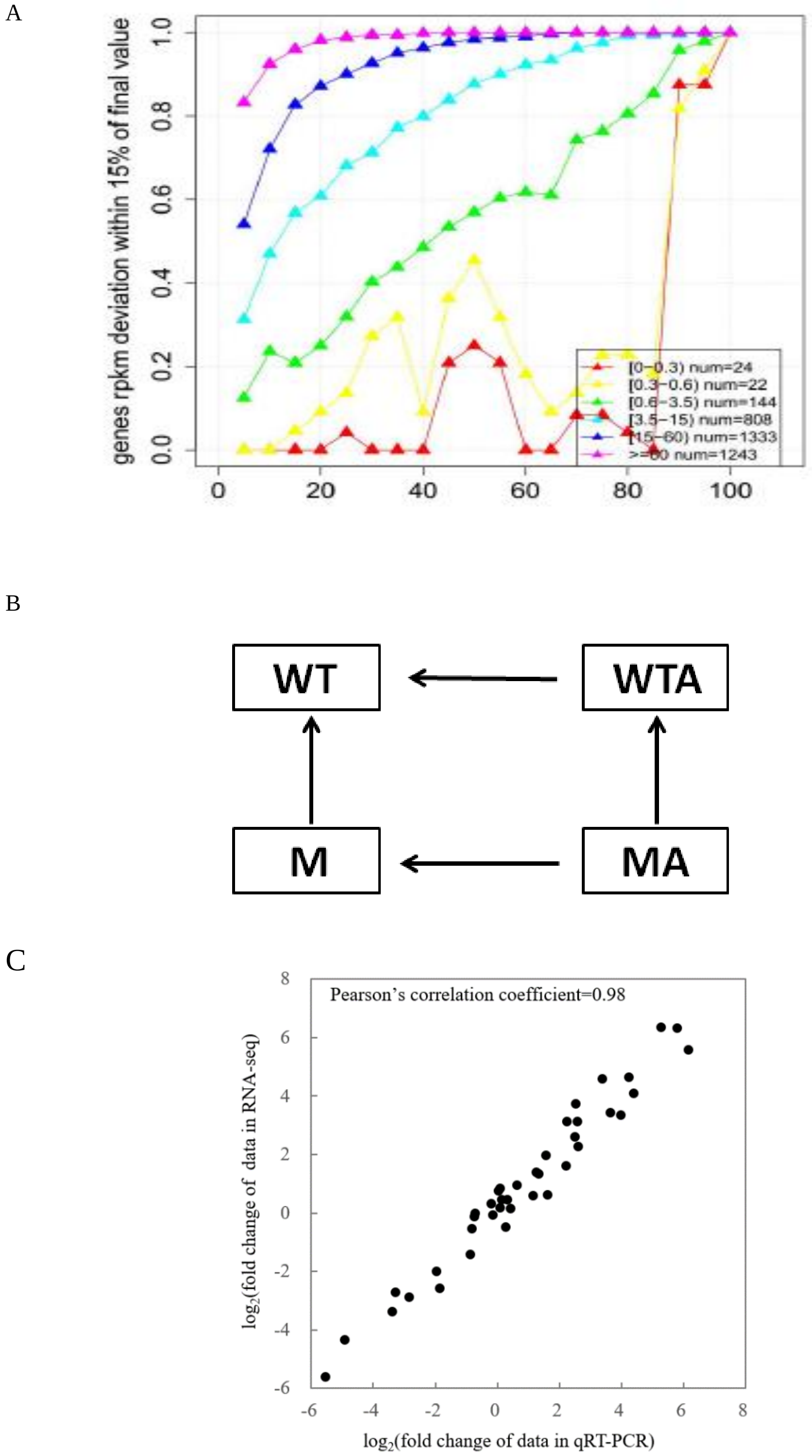


**Supplemental Figure S2. RNA sequencing data analysis and validation.** (A) Representative saturation curve of sample M1. (B) Comparison scheme of differential expressed genes between each two samples pairs indicated as arrow. WT: wild type in $Na_2SO_4$; M: △*slr1821* knockout mutant in $Na_2SO_4$; WTA: wild type in $(NH_4)_2SO_4$; MA: △*slr1821* knockout mutant in $(NH_4)_2SO_4$. The arrow indicated comparison direction, for example, MA/M. (C) Validation of sequencing data by quantitative RT-PCR for representative genes.

**A**

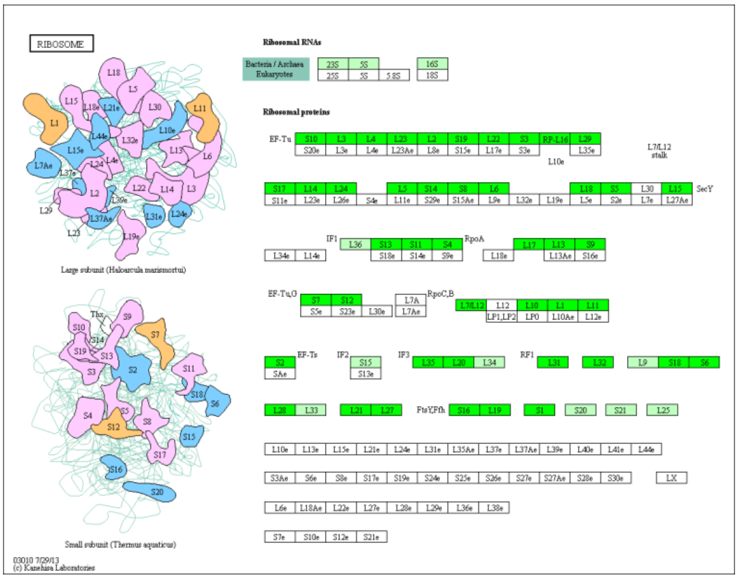


**B**

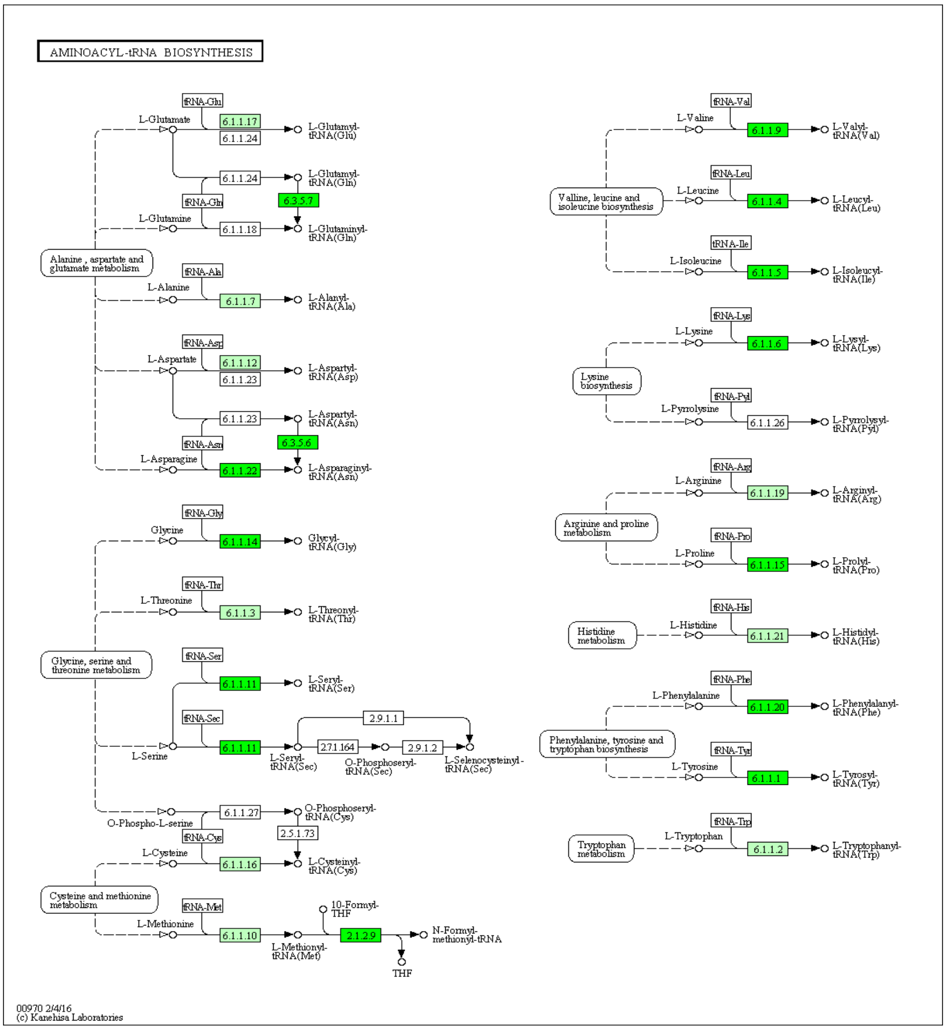


**Supplemental Figure S3. Suppressed expression of translational machinery in △*slr1821* vs WT upon $NH_4^+$ stress.** (A) Ribosomal proteins in ribosome large and small subunit. (B) Synthetase involved in aminoacyl-tRNA biosynthesis. Dark green highlights the suppressed gene, light green is the component without significant regulation. White ones were components not available in Synechocystis 6803.

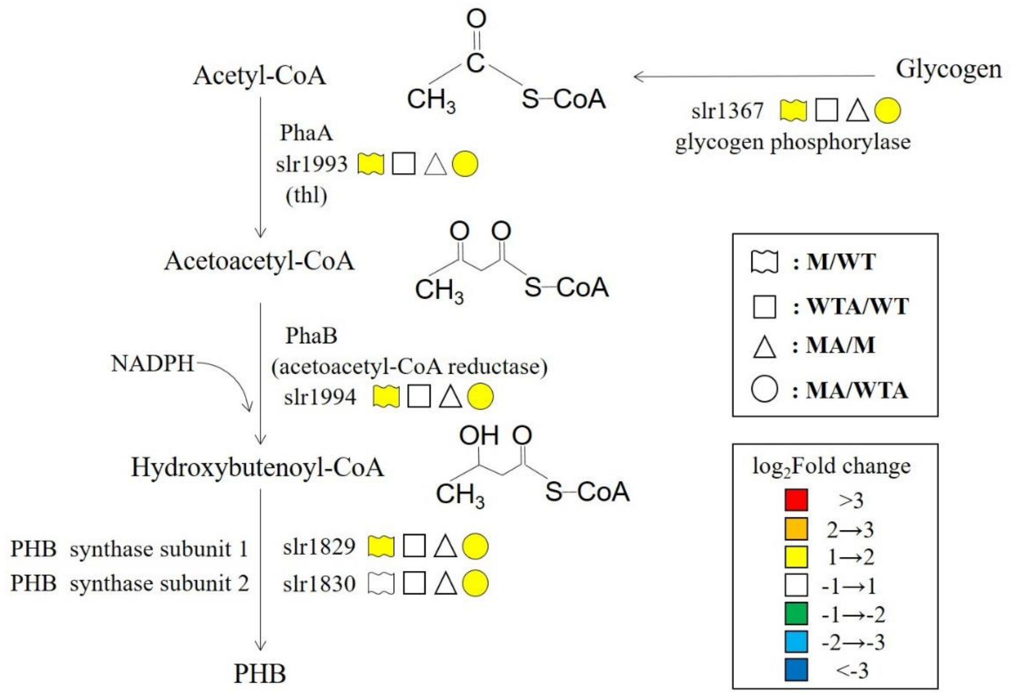


**Supplemental Figure S4. Knockout of *slr1821* resulted in higher expression of PHB synthesis genes.** The comparison pair and fold changes are indicated in different shapes and colors respectively. WT and WTA are wild type without and with $NH_4^+$ stress. M and MA are Δ*slr1821* knockout mutant without and with $NH_4^+$ stress.
